# Rice husk and melaleuca biochar additions reduce soil CH _4_ and N _2_O emissions and increase soil physicochemical properties

**DOI:** 10.12688/f1000research.74041.2

**Published:** 2022-02-15

**Authors:** Nam Tran Sy, Thao Huynh Van, Chiem Nguyen Huu, Cong Nguyen Van, Tarao Mitsunori

**Affiliations:** 1 College of Environment and Natural resources, 3/2 street, Can Tho University, Can Tho city, 900000, Vietnam; 2 Faculty of Agriculture, Tokyo University of Agriculture and Technology, Tokyo, 183-8506, Japan

**Keywords:** Biochar amendment, conventional rice farming, greenhouse gas emissions, melaleuca biochar, rice-husk biochar, soil fertility

## Abstract

**Background**: Biochar is a promising material in mitigating greenhouse gases (GHGs) emissions from paddy fields due to its remarkable structural properties. Rice husk biochar (RhB) and melaleuca biochar (MB) are amendment materials that could be used to potentially reduce emissions in the Vietnamese Mekong Delta (VMD). However, their effects on CH _4_ and N _2_O emissions and soil under local water management and conventional rice cultivation have not been thoroughly investigated.

**Methods**: We conducted a field experiment using biochar additions to the topsoil layer (0-20 cm). Five treatments comprising 0 t ha ^-1^ (CT0); 5 t ha ^-1^ (RhB5) and 10 t ha ^-1^ (RhB10), and 5 t ha ^-1^ (MB5) and 10 t ha ^-1^ (MB10) were designed plot-by-plot (20 m ^2^) in triplicates.

**Results**: The results showed that biochar application from 5 to 10 t ha ^-1^ significantly decreased cumulative CH _4_ (24.2-28.0%, RhB; 22.0-14.1%, MB) and N _2_O (25.6-41.0%, RhB; 38.4–56.4%, MB) fluxes without a reduction in grain yield. Increasing the biochar application rate further did not decrease significantly total CH _4_ and N _2_O fluxes but was seen to significantly reduce the global warming potential (GWP) and yield-scale GWP in the RhB treatments. Biochar application improved soil Eh but had no effects on soil pH. Whereas CH _4_ flux correlated negatively with soil Eh ( *P < *0.001; *r ^2 ^* = 0.552, RhB; *P < *0.001; *r ^2 ^* = 0.502, MB). Ameliorating soil aeration and functions by adding RhB and MB resulted in improving soil physicochemical properties, especially significant SOM and AN boosting, which indicate better soil health, structure, and fertility.

**Conclusions**: Biochar supplementation significantly reduced CH _4_ and N _2_O fluxes and improved soil mineralization and physicochemical properties toward beneficial for rice plants. The results suggest that the optimal combination of biochar-application rates and effective water-irrigation techniques for soil types in the MD should be further studied in future works.

## Introduction

In Vietnam, the agricultural sector contributes approximately 30% of national greenhouse gases (GHGs) emissions ( MONRE, 2017). For rice cultivation, paddy fields are the primary source of GHGs emissions ( Nan *et al.*, 2020; Shinoda *et al.*, 2019), accounting for 50% of the sub-sectors in agricultural production and roughly 14.6% of national GHG emissions in Vietnam ( MONRE, 2017). According to NDC (2020), Vietnam is committed to reducing 8% of total national GHGs emissions from domestic resources by 2030. Management and technological strategies will play a vital role in reducing the total carbon footprint. Biochar is a carbonized biomass product produced from thermochemical conversion of organic materials under oxygen-limited conditions ( Lohri *et al.*, 2016; Wu *et al.*, 2012; Waqas *et al.*, 2018). Biochar applications have been noted as one of the most promising approaches for reducing GHGs emissions from rice production ( Koyama *et al*., 2015; Wu *et al.*, 2019a; *Nan et al.*, 2021), and IPCC recently recommended the method ( Ji *et al.*, 2020). Previous studies have demonstrated that biochar incorporated into soil paddy fields positively rehabilitated soil properties such as pH neutralization, cation exchange capacity (CEC), and buffering capability, soil organic materials (SOM), and nitrogen storage ( Qin *et al.*, 2016; Luo *et al.*, 2020); improved plant available water, microporosity, and soil aggregate stability, and decreased bulk density ( Burrell *et al.*, 2016); effected on soil functions and fertility ( Giagnoni *et al.*, 2019; Siedt *et al.*, 2021); and ameliorated nutrient availability of carbon (C), nitrogen (N), phosphorus (P), potassium (K), magnesium (Mg) and Calcium (Ca) ( Li *et al.*, 2019). Furthermore, biochar forms a great habitat for different microorganisms via providing macro-, meso- and micropores ( Palansooriya *et al.*, 2019; Wu *et al.*, 2019a), supports microbial communities by providing labile C substrates for degradation ( Smith *et al.*, 2010), stimulating biodiversity and abundance of methanotrophic microbes ( Qin *et al.*, 2016). Moreover, the addition of biochar to the soil reduces GHGs emissions ( Spokas and Reicosky, 2009; Koyama *et al.*, 2015; Nan *et al.*, 2020; Huang *et al.*, 2019) and increases rice yield under different favorable conditions ( Yang *et al.*, 2019; Paiman and Effendy, 2020).

In the Vietnamese Mekong Delta (VMD), melaleuca is an abundantly available hard firewood resource, accounting for 176,295 ha ( GIZ, 2009); the wood reserve of melaleuca is estimated at 13 million m ^3^. In addition, rice husk is known as a by-product of rice production, accounting for 20% of rice yields ( Chungsangunsit *et al*., 2009). It is estimated that VMD annually produces around 1.9 million tons of rice husk ( Son *et al.*, 2017). Biomass (hardwood and crop residues) are often used as typical feedstock for making biochar pyrolysis owing to their multiple-porous structure ( Nguyen *et al.*, 2018; Nan *et al.*, 2020), which facilitates the multifunctional purposes of soil amendment and pollutant remediation. Therefore, both melaleuca and rice husk could be used to produce biochar, which is then applied to rice paddy fields as a GHGs emission reduction strategy. Although previous studies have demonstrated the effectiveness of biochar incorporation on reducing GHGs emissions, little attention has been paid to the quantitative variation of rice husk biochar (RhB) and melaleuca biochar (MB) on GHGs emissions and soil improvement in VMD lowland conditions. Moreover, the majority of previous studies exclusively emphasize CH _4_ and N _2_O emissions on water practices by controlled irrigation, and alternative wetting and drying, and midseason drainage ( Yang *et al.* 2019, Sriphirom *et al.* 2020, Uno *et al.* 2021), while atypical water irrigation regime has not been thoroughly elucidated.

Thus, we aimed (i) to elucidate the CH _4_ and N _2_O emissions and global warming potential (GWP) from the incorporation of RhB and MB into the paddy field soils under locally typical water management regimes in the VMD, and (ii) to determine the effects of RhB and MB amendments on soil physicochemical properties. We, therefore, conducted a field experiment with a variety of RhB and MB amendment amounts under conventional farming practices. Our field experimentation confirmed that RhB and MB application to rice paddy fields was feasible in reducing GHGs emissions. Simultaneously, biochar application improved soil availability of SOM and anaerobically mineralized N.

## Methods

### Site description

A field experiment was carried out on a typical smallholding farmer's paddy field in Thoi An Dong Village, Can Tho city, Vietnam (10°3′44″N, 105°41′55″E). The study area was located in the center of the Mekong Delta, Vietnam, which is a tropical area influenced by the monsoon climate zone, with measured mean annual rainfall (2,088.4 mm), air temperature (27.5 °C), humidity (78.0 – 86.0%), sunshine (2,467.4 – 2,695.4 hours) in the period from 2015-2019 ( DONRE, 2020). The precipitation and temperature during the experiment were recorded by a weather station placed at the farmer's house (~150 m from the field experiment). The soil was classified as Thionic Glycesol (International Union of Soil Sciences (IUSS) working group World Reference Base (WRB), 2015) ( Dong *et al.*, 2012, Minamikawa *et al.,* 2021). The elementary properties were (0-20 cm depth) as follows: pH (H _2_O), 5.41; EC, 0.9 mS cm ^−1^; bulk density, 0.92 g.cm ^−3^, silty clay texture (59.3% clay, 39.5% silt, 1.2% sand); organic matter, 87 g kg ^−1^; total N, 4.21 g kg ^−1^; cation exchange capacity (CEC), 37.4 meq 100 g ^−1^; exchangeable K, 0.54 meq 100 g ^−1^, exchangeable Mg, 5.47 meq 100 g ^−1^; exchangeable Ca, 10.5 meq 100 g ^−1^; and total C, 40.76 g kg ^−1^.

### Biochar preparation

RhB was made on-site using a simple semi-industrial pyrolysis batch method ( Oikawa *et al*., 2016). Here a short iron bar was to set onto the ground. A stainless chimney pipe 1.5m long was vertically erected to the bar using wire. The pipe was kept at a 10-cm distance from the ground to release smoke generated during the pyrolysis process. Embers were placed adjacent to the bar to kick off the carbonization process. Then, rice husk was poured around the bar according to a coniform shape with 1.5 m height and 1.5 m diameter. RhB was generated from the bottom to the summit. After finishing the pyrolysis process (two days), RhB was watered to achieve ambient temperature.

**Figure 1.**
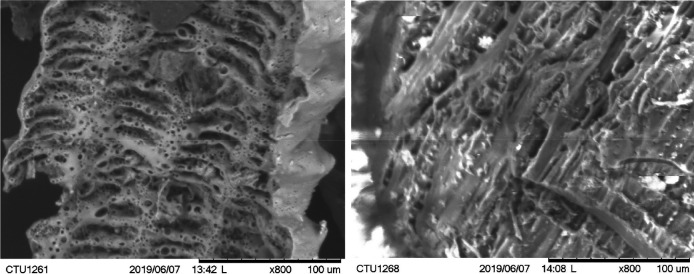
Scanning electron microscope (SEM) images of biochar produced from rice husk (a) and melaleuca (b) at X800 magnification.

MB was produced by a poor-oxygen pyrolysis process under a traditional bell-shaped charcoal production kiln for a 30-day batch. The kiln was made from baked bricks, clay, and sand mortar. The kiln’s structure comprises a bell-shaped heating firewood chamber, a door used for firewood loading, and biochar unloading. A combustion chamber provided hot air for the carbonization process, while four chimneys were installed around the heating chamber discharging smoke during the carbonization process. Firewood was fully loaded according to each layer inside the heating chamber; the lowest layer was kept 10 cm away from the ground to ensure air convection. Before starting, the door was closed to begin the carbonization process. Air heating from the combustion chamber was slowly provided to the inner heating chamber to form carbonization. After 30 days of pyrolysis, the heating was switched off, and the combustion chamber was blocked off for an additional 15 days to cool to ambient temperature. The images of RhB and MB and their properties are shown in Figure 1 and Table 1, respectively.

**Table 1.** Main properties of biochar derived from rice husk and melaleuca used in the field experiment.

Items	Rice husk	Melaleuca
pH (H _2_O)	9.56	7.54
EC (mS cm ^−1^)	0.78	0.28
CEC (cmol (+) kg ^−1^)	13.2	9.55
Total C (g kg ^−1^)	253.5	291.8
Total N (g kg ^−1^)	3.26	2.50
Total P (g kg ^−1^)	0.13	0.33
Specific surface area (m ^2^ g ^−1^)	51.93	2.04
Total pore volume (cm ^3^ g ^−1^)	0.026	0.001

### Experimental design

The size of each experimental plot was 20 m ^2^ (4 m × 5 m) which were arranged in a randomized complete block design with three replications. Each plot was separated by soil banks and covered with mulch film. Five treatments with RhB and MB incorporated into the soil paddy field comprised 0 t ha ^−1^ (conventional rice cultivation without biochar supplementation), 5 t ha ^−1^, and 10 t ha ^−1^ (based dried weight) named CT0, RhB5, RhB10, MB5, and MB10, respectively. Biochar was manually spread on the soil surface of each pot and evenly incorporated into the plow layer of soil (approximately 20 cm) by shovels and rakes before sowing. Biochar additions were applied one time solely at the beginning of the experiment.

### Rice cultivation and water management

According to the local crop calendar, the experiment time corresponded with the Spring-Summer (SS) season (the second crop) ( Table 2). This is a transitional season between the dry and wet seasons. Rice straw and rice stubble from the previous rice crop cycle (Winter-Spring) were plowed by a hand tractor and underwent a 7-day fallow period before sowing. A short-duration variety of rice (IR50404 cultivar) typically grown in VMD was used in this field experiment (85-90 days of maturity). Pre-germinated seeds were sown on the wet-leveled soil using drum seeders at a rate equivalent to 120 kg ha ^−1^. The irrigation followed regionally typical water management based on the farmer’s practical experience.

**Table 2.** Rice cropping calendar in the field experiment.

Cultivated schedule	Date of experiment ^1)^	Days after sowing
Plowing	14/03/2019	−7
Biochar incorporation	21/03/2019	0
Sowing	21/03/2021	0
Starting irrigation	29/03/2021	8
Fertilization		
1 ^st^ topdressing (16-8-20) ^2)^	30/03/2019	9
2 ^nd^ topdressing (32-16-0) ^2)^	13/04/2019	23
3 ^rd^ topdressing (32-16-20) ^2)^	27/04/2019	38
Drainage	30/05/2019	70
Harvest	14/06/2019	85

^1)^ dd/mm/yyyy.

^2)^ The numbers in parenthesis indicate the amount (kg ha ^−1^) of fertilizers applied in terms of N, P and K, respectively.

### Fertilizer application

Inorganic fertilizers with the total amount of 80 kg N ha ^−1^, 40 kg P _2_O _5_ ha ^−1^ and 40 kg KCl ha ^−1^ were applied. The fertilization was divided into intervals at 9, 23, and 38 days after sowing (DAS) by broadcasting. Nitrogen (N) was applied as urea at a rate of 16-32-32 kg N ha ^−1^ (broadcasted three times). Phosphorus (P) was applied as superphosphate at a rate of 8-16-16 kg P _2_O _5_ ha ^−1^ tolerant (broadcasted three times). Whereas potassium (K) was applied as potassium chloride at a 20-0-20 kg KCl ha ^−1^ rate (broadcasted twice). The rice cropping calendar and fertilizer application are shown in Table 2.

### Measurements

Scanning electron microscope (SEM) images of RhB and MB were captured by microscope (TM-1000, Hitachi, Japan). Specific surface area and total pore volume were determined using BET Surface Area Analyzer (Quatachrome Nova 1000e, USA).

A weather station (WS-GP1, Delta-T Devices, Cambridge, UK) was installed on-site to record hourly temperature and rainfall at the experimental site. Redox potential (Eh) at plow-layer soil (20 cm) was measured by using platinum-tipped electrodes pined into the ground at a depth of 5, 10, and 20 cm; a portable Eh meter (HM31P; TOA-DKK, Japan) was connected to the electrodes to record soil Eh values at corresponding times to gas sampling. Surface water levels were also recorded simultaneously with gas sampling, using a ruler to read values directly in a plastic-perforated tube pre-installed in each plot.

Topsoil samples (0-20 cm) in each plot were collected before adding biochar and harvest by an auger 3 cm diameter. Visible remaining biomass was eliminated before air drying and sieved at 2.0 mm. Initial soil samples ( *n =* 15) were mixed into a collective sample for analysis. Harvest soil samples were collected for each plot separately. Physical soil properties were measured as follows: soil texture - Pipette Robinson method ( Carter and Gregorich, 2008), bulk density - Core method, and the particle density of soil ( Blake and Hartge, 1986). Biochar and soil chemical properties were detected as follows: pH (H _2_O) – a portable pH meter (HANA, Germany), soil organic matter (SOM) and total organic C (TOC) – Walkley and Black (1934), total P - Bowman (1988), available P (AP) - Olsen and Sommers (1982), total N – semi-micro Kjeldahl method ( Bremner, 1996), anaerobically mineralized N (AN)– a 7-day anaerobic incubation at 40 °C ( Keeney and Bremner, 1996), and CEC and exchangeable cations – Thomas (1982).

Rice yield was determined by harvesting from a 2.5 m × 2.0 m area in each plot at physiological maturity and removed unfilled grains by water before sun drying. A grain moisture tester (Riceter f2, Kett Electric Laboratory, Tokyo, Japan) was used to measure moisture content. The presented rice yield was adjusted to a 14% moisture content.

The closed chamber method was used to collect gas samples. A chamber was made from transparent polyvinyl chloride (PVC) panels with a 1.5 mm thickness. The cross-sectional area was 0.25 m ^2^ (0.5 m × 0.5 m). The height of the chamber was 70 cm from the bottom to the top layer. The chamber inside was equipped with a circulating fan, a temperature meter, and a pressure control plastic bag as described in detail by Minamikawa (2015). The chamber was placed on a plastic pre-installed base (a groove 4.5 cm depth) in each plot and sealed off by water before sampling. After chamber closure, a syringe (50 mL) was utilized to take inside gas at 1, 11, 21, and 31 minutes. Then, gas samples were injected into a 20-mL evacuated vial. The gas sampling was carried out from 10 DAS to 73 DAS at 7-day intervals. The concentrations of CH _4_ and N _2_O were analyzed with a gas chromatograph (8610C, SRI Instruments, CA, USA) equipped with a flame ionization detector (FID) and an electron capture detector (ECD) for the analysis of CH _4_ and N _2_O, respectively. The columns for the analysis of CH _4_ and N _2_O were packed with Porapak Q (50–80 mesh); dinitrogen (N _2_) was used as the carrier gas for both FID and ECD.

Porosity was calculated by dividing volume pores (based on the subtraction between bulk density and particle density of soils) by volume total ( Flint and Flint, 2002). CH _4_ and N _2_O fluxes were calculated by a linear progression of gas concentration change over time, and total fluxes of CH _4_ and N _2_O were calculated using a trapezoidal integration method described by Minamikawa (2015). Global warming potential (GWP) was calculated based on CO _2_ equivalence (1 CH _4_ = 34 CO _2_-eq; 1 N _2_O = 298 CO _2_-eq) at a 100-year scale of climate-carbon feedbacks ( Myhre *et al.*, 2013). Yield-scale GWP was calculated by dividing the GWP by grain yield ( Minamikawa *et al.*, 2021).

### Statistical analysis

One-Way analysis of variance (ANOVA) was used to assess the effects of each biochar on grain yield, gas fluxes, GWP, yield-scale GWP, and soil improvement. The difference of treatments was carried out using Duncan’s method for all pairwise multiple comparison procedures. Linear regression analyses were performed to assess the relationship between Eh change and methane emission. We also analyzed the relationship between biochar application rate and gas emissions. In the statistical analysis, we did not compare the difference between RhB and MB. All analyses were carried out using R stats Version 4.2.0 (R Project for Statistical Computing, RRID:SCR_001905). The results are presented in tabular form with the values including mean ± standard deviation (SD) and the different symbols with a confidence level of 95%. Significant different comparison among treatments was considered at Duncan’s multiple range test (*** *P* < 0.001, ** *P* < 0.01, * *P* < 0.05 and † *P* > 0.05) after passing homogeneity of variance.

## Results

### Weather and water management

**Figure 2.**
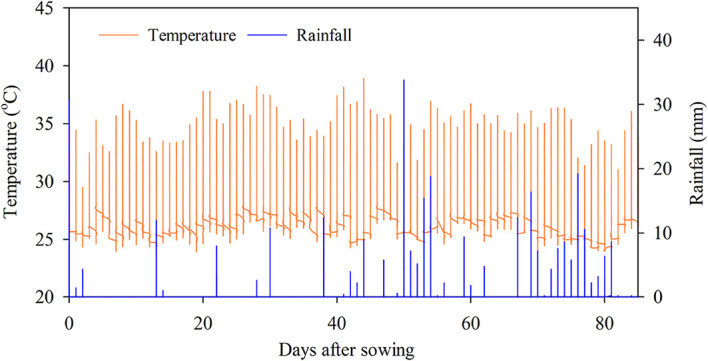
Temperature and rainfall during the field experiment.

**Figure 3.**
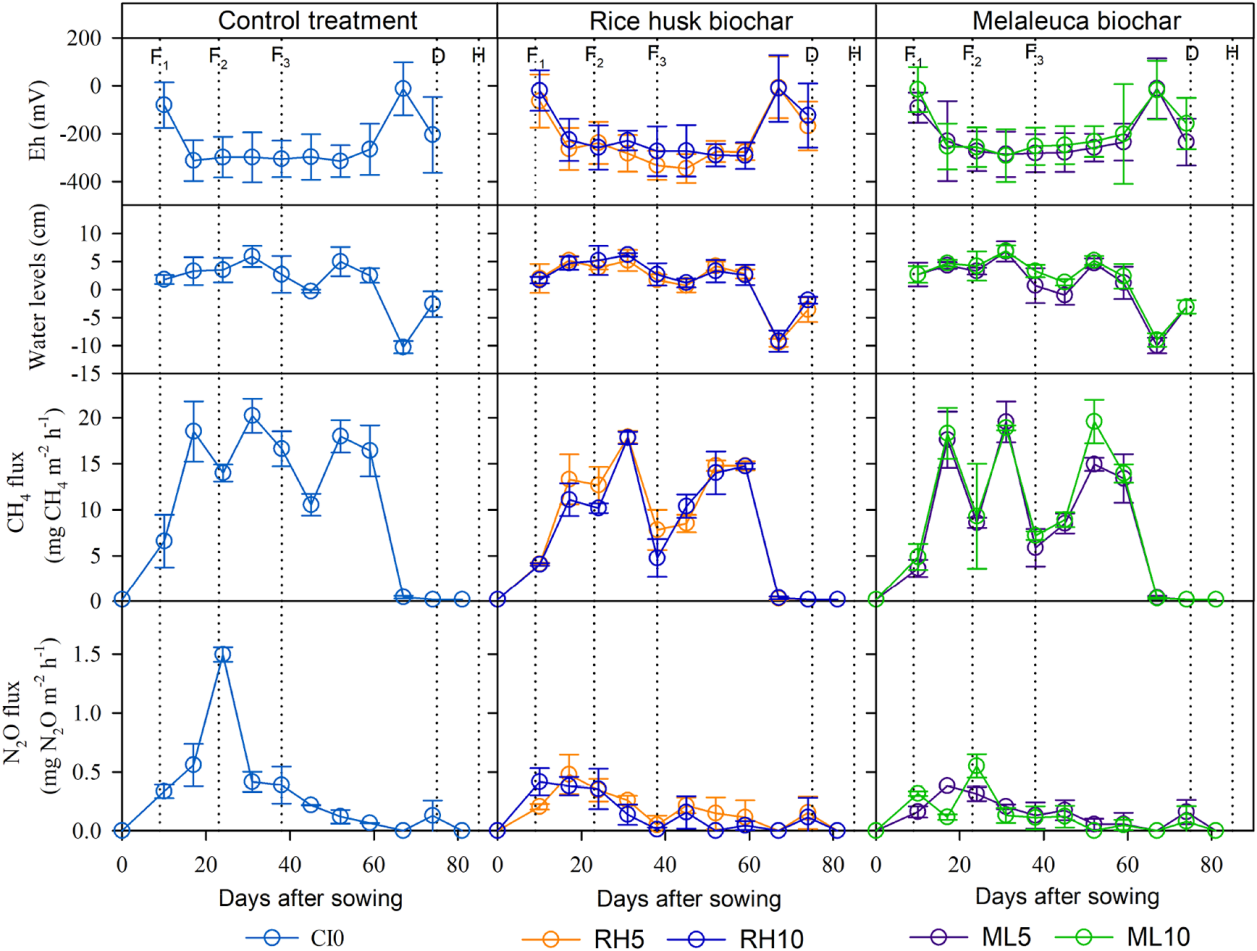
Time course changes in soil redox potential (Eh), water level, hourly CH _4_ and N _2_O fluxes in the paddy field applied without (left) or with RhB (center) or MB (right) during the field experiment. Error bars indicate the standard error ( *n* = 3). Vertical dotted lines illustrate agronomic management of the first, the second and the third topdressing fertilizer (F _1_, F _2_ and F _3_, respectively), drainage (D) and harvest (H).

The mean air temperature and the total rainfall during the experiment were 28.9 °C and 429 mm, respectively ( Figure 2). High rainfall was observed between 40-60 and 65-80 DAS. Figure 3 shows that the flooding water regime was predominantly observed during the experimental regression. The trend of water levels variation was similar over treatments. Water was irrigated from 7 DAS, reflooded 3-5 cm from soil surface for fertilizing (9, 23, and 38 DAS), and respective multiple drainage practices (−10 to 5 cm) ( Uno *et al*., 2021) was carried out for the remaining periods. Fifteen days before harvesting (70 DAS), the soil was drained and kept saturated to minimize rice lodging and easy-to-harvest grain. Rice plants flowered and headed during 45-60 DAS.

### CH _4_ and N _2_O emissions

**Figure 4.**
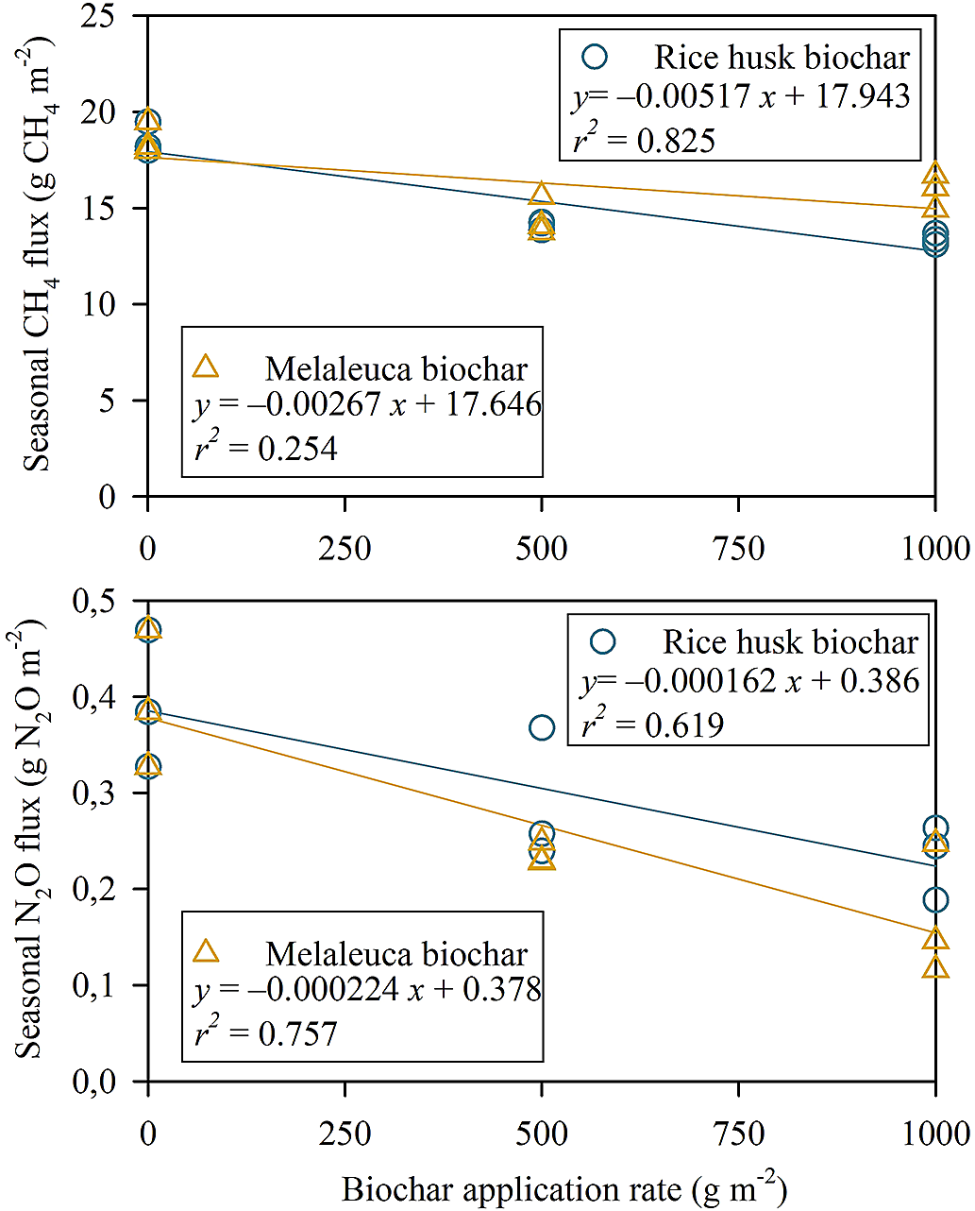
Relationship between biochar application rate and total CH _4_ (above) and N _2_O (below) fluxes during the field experiment. Each symbol represents one replication in each treatment.

CH _4_ emissions gradually increased in the early rice growth stage (0-17 DAS) and almost stopped after drainage (70 DAS) ( Figure 3). It should be noted that CH _4_ flux was predominant in the period from 17 – 59 DAS and several CH _4_ flux peaks were observed between treatments (i.e., three peaks were observed in MB5 and MB10). Maximum CH _4_ flux peaks were reached simultaneously in all treatments after 31 DAS. Highest peaks between treatments are represented in a descending way as follows: CT0 > MB5 > MB10 > RhB5 > RhB10. Compared to the CT0 treatment, biochar application reduced total CH _4_ emissions significantly ( Table 3). Particularly, RhB5 and RhB10 mitigated total CH _4_ flux from 24.2 to 28.0%, respectively, while MB5 and MB10 alleviated between 22.0 and 14.1%, respectively. Irrespective of RhB and MB, the CH _4_ flux was insignificant with an increasing biochar addition rate from 5 to 10 t ha ^−1^ ( *P* < 0.01). There was a negative linear regression relationship between biochar application rate and total CH _4_ emission ( *P <* 0.001, *r* ^2^ = 0.825) ( Figure 4). In contrast, the linear regression of melaleuca biochar was poorly explained with increasing biochar amendment rate and total CH _4_ flux ( *P =* 0.095, *r* ^2^ = 0.254).

**Table 3.** Grain, total CH _4_ and N _2_O fluxes, global warming potential (GWP) and yield-scaled GWP at 100 years scale in the paddy field applied without or with biochar ^1)^.

Treatment ^2)^	Grain (g m ^−2^)	CH _4_ (g CH _4_ m ^−2^)	N _2_O (g N _2_O m ^−2^)	GWP (g CO _2_-eq m ^−2^)	Yield-scaled GWP (g CO _2_-eq m ^−2^)
CT0	498 ± 47.6	18.6 ± 0.80 ^aA^	0.39 ± 0.07 ^aA^	749 ± 13.5 ^aA^	1.51 ± 0.13 ^aA^
RhB5	513 ± 56.2	14.1 ± 0.23 ^b^	0.29 ± 0.07 ^ab^	566 ± 25.5 ^b^	1.12 ± 0.18 ^b^
RhB10	510 ± 33.0	13.4 ± 0.30 ^b^	0.23 ± 0.04 ^b^	524 ± 2.76 ^c^	1.03 ± 0.06 ^c^
MB5	519 ± 9.86	14.5 ± 1.00 ^B^	0.24 ± 0.01 ^B^	563 ± 31.6 ^B^	1.09 ± 0.08 ^B^
MB10	517 ± 10.9	15.9 ± 0.90 ^B^	0.17 ± 0.07 ^B^	591 ± 10.8 ^B^	1.14 ± 0.44 ^B^
*P* value ^3)^					
CT × RhB	†	***	*	***	**
CT × MB	†	**	**	***	**

^1)^ Data represent as means ± SD ( *n* = 3).

^2)^ CT0, control treatment; RhB5 and RhB10, 5 and 10 t ha ^−1^ rice-husk biochar amendment, respectively; MB5 and MB10, 5 and 10 t ha ^−1^ melaleuca biochar amendment, respectively.

^3)^ Statistical analysis did not compare between RhB and MB. The letters indicate significant difference according to Duncan’s multiple range test (*** *P* < 0.001, ** *P* < 0.01, * *P* < 0.05 and † *P* > 0.05). Normal and capital lowercases indicate a significant difference between CT0 vs. RhB and CT0 vs. MB, respectively.

N _2_O was released mainly in the early stage of rice growth in all treatments ( Figure 3). The highest N _2_O flux peaks were observed in the CT0 (24 DAS). All measured values were below 1.5 mg N _2_O m ^−2^ h ^−1^. As observed, N _2_O flux flushed mainly during the fertilizing period from 9 to 38 DAS, even though experimental pots were predominantly flooded, especially in the CT0 accounted for 56.8% in total, while RhB and MB varied by 50.6-53.1% and 52.3-47.6%, respectively. Total N _2_O emission was reduced in RhB or MB applied soil compared to CT0 ( Table 3). Specifically, RhB10 significantly reduced by approximately 41.0%, whereas MB5 and MB10 by 38.5 and 56.4%, respectively. However, the reduction of total N _2_O flux was insignificant in MB5. As a result, there were different negative linear relationships of biochar application rate and total N _2_O flux (RhB, *P =* 0.012, *r* ^2^ = 0.619; MB, *P =* 0.002, *r* ^2^ = 0.757) ( Figure 4).

### Rice yield, GWP, and yield-scaled GWP

Biochar addition to the soil slightly increased rice yield compared to the CT0, but the statistical analysis was insignificant ( Table 3). A similar pattern about emissions was seen among GWP, yield-scaled GWP, and total CH _4_ flux due to CH _4_ flux was greatest contribute to GWP, yield-scaled GWP. The RhB additions significantly decreased the GWP and yield-scaled GWP by 24.4 – 30.0% and 25.8 – 31.8% for RhB5 and RhB10, respectively. Although MB significantly diminished the GWP and yield-scaled GWP by 24.8 – 21.09% and 27.8 – 24.5%, respectively, there was no significant difference between MB5 and MB10.

### Soil characteristics

**Figure 5.**
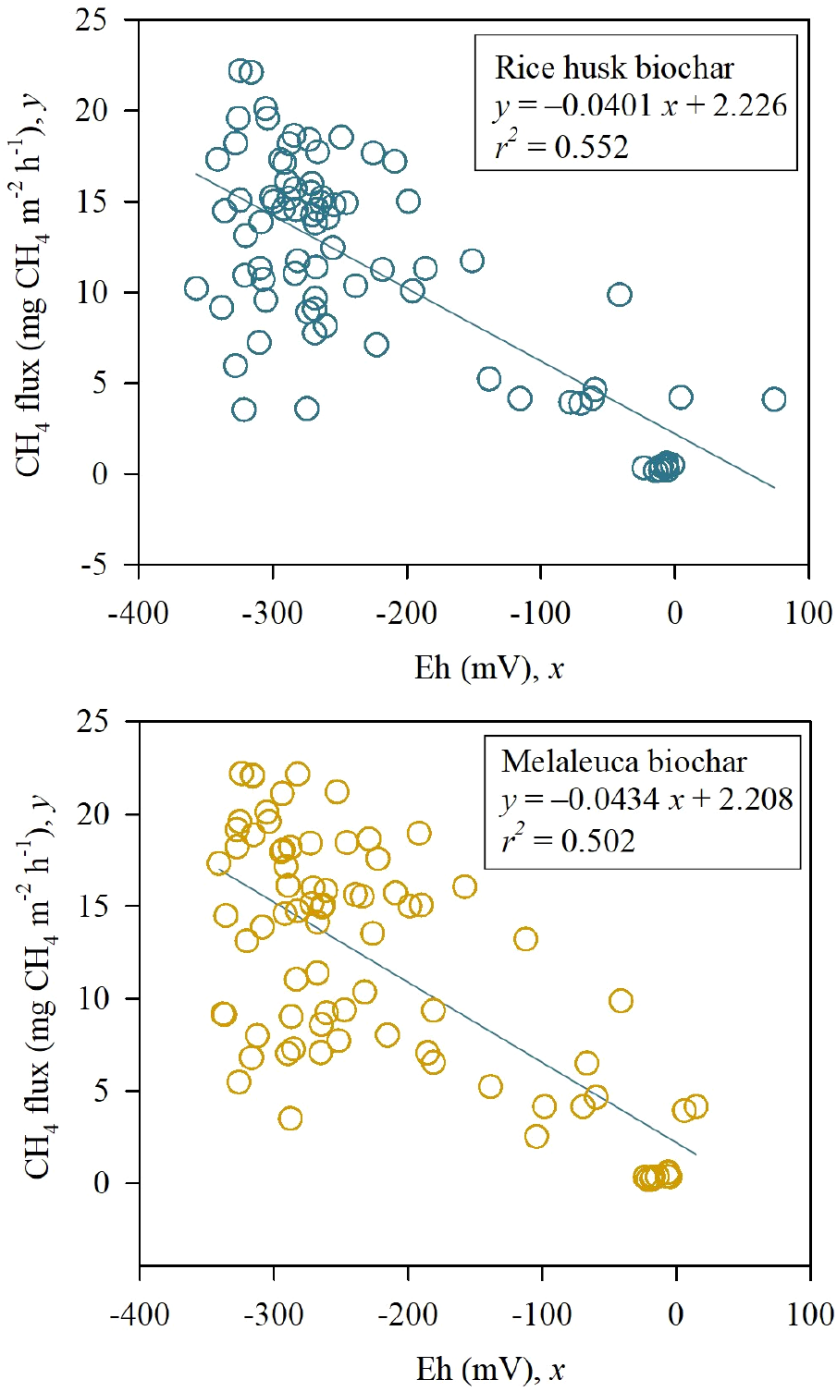
Relationship between the hourly CH _4_ flux and Eh in the field applied with RhB (above) or MB (below).

A similar performance pattern of soil Eh condition was seen among treatments ( Figure 3). Eh reduced after initial irrigation and was seen to reach a stable level (below -250 mV) during the rice growth period from 17 to 66 DAS. Whereas the final drainage rapidly increased the soil Eh condition (73 DAS) in all treatments. The supplementation of RhB and MB obviously improved soil Eh condition compared to the CT0 by 7.44 – 14.5% and 10.7 – 19.0%, respectively ( Table 4). There was a negative linear relationship between hourly CH _4_ flux and the Eh values in RhB ( *P <* 0.001; *r* ^2^ = 0.552) and MB ( *P <* 0.001; *r* ^2^ = 0.502) ( Figure 5).

**Table 4.** Physiochemical properties ^1)^ of soil applied without or with biochar.

Treatment ^2)^	pH	Eh ^3)^ (mV)	Bulk density (g cm ^−3^)	Porosity (%)	SOM (g kg ^−1^)	AP (mg kg ^−1^)	AN (mg kg ^−1^)
CT0	4.69 ± 0.10	-242 ± 12.3 ^cB^	0.97 ± 0.10 ^aA^	53.3 ± 0.75 ^bB^	31.8 ± 0.36 ^cC^	19.0 ± 3.95 ^B^	10.7 ± 1.03 ^bB^
RhB5	4.81 ± 0.21	-224 ± 6.12 ^b^	0.78 ± 0.04 ^b^	61.5 ± 7.20 ^ab^	43.2 ± 2.08 ^b^	21.8 ± 3.48	15.5 ± 0.52 ^a^
RhB10	5.26 ± 0.64	-207 ± 2.13 ^a^	0.74 ± 0.13 ^b^	65.1 ± 2.07 ^a^	47.6 ± 1.13 ^a^	25.2 ± 4.04	14.8 ± 0.60 ^a^
MB5	4.82 ± 0.13	-216 ± 16.3 ^A^	0.75 ± 0.06 ^B^	55.5 ± 1.92 ^A^	39.1 ± 3.03 ^B^	25.1 ± 2.81 ^AB^	14.5 ± 1.10 ^AB^
MB10	4.68 ± 0.17	-196 ± 5.78 ^A^	0.71 ± 0.05 ^B^	62.9 ± 4.19 ^A^	45.0 ± 1.30 ^A^	28.8 ± 2.79 ^A^	16.6 ± 2.97 ^A^
*P* value ^4)^							
CT × RhB	†	*	*	*	***	†	***
CT × MB	†	*	***	*	***	*	*

^1)^ Data represent as means ± SD ( *n* = 3).

^2)^ Abbreviations are the same as Table 3.

^3)^ Mean value is based on the whole values measured during the experimentation in each plot at 3 soil levels depth comprising 5, 10 and 20 cm between 10 and 64 DAS.

^4)^ Statistical analysis was carried out as the same as Table 3.

Table 4 represents the soil characteristic differences between treatments at the time of harvest. Overall, although biochar amendment was seen to increase soil pH slightly, statistical analysis implied no significant difference between treatments. Yet, biochar amendment significantly reduced the soil bulk density (RhB5, 19%; RhB10, 23%; MB5, 22.7% and MB10 26.8%) and ameliorated the soil porosity (RhB5, 8.2%; RhB10, 11.8%; MB5, 2.2%, and MB10 9.6%). However, increasing RhB and MB biochar application rate from 5 to 10 t ha ^−1^ did not significantly change soil bulk density and porosity. Moreover, intensifying biochar incorporation significantly increased SOM by 38.6 – 52.7% for RhB and 25.4 – 45.9% for MB. Notably, AN in biochar-applied treatments was higher than that of the CT0 by 44.8 – 38.3% and 35.5 – 55.1% for RhB and MB, respectively. AP significantly increased in the MB treatments by 32.1 – 51.58% but did not in RhB. Although additional biochar increased the available and mineralized nutrients, statistical analysis results showed no significant difference between biochar application rates of 5 to 10 t ha ^−1^ ( Table 4) ( Tran Sy *et al*., 2021).

## Discussion

### Effects of biochar incorporation on CH _4_ and N _2_O fluxes

Conventional practices without biochar application released 18.6 g CH _4_ m ^−2^ and 0.39 g N _2_O m ^−2^ ( Table 3). These values are in accordance with previous findings conducted in the VMD ( Vo *et al.* 2020; Minamikawa *et al.* 2021; Uno *et al.* 2021). Notably, RhB and MB amendments under typically local water management, and conventional practices significantly reduced CH _4_ flux by 24.2 % in RhB5, 28.0 % in RhB10, 22.0 % in MB5, 14.1 % in MB10 and N _2_O flux by 38.5 % in RhB5, 56.4 % in RhB10, 25.6 % in MB5, 41.0 % in MB10, and slightly improved rice yield (2.41-4.21%) ( Table 3). Similarly, Yang *et al.* (2019) demonstrated that biochar additions (20 - 40 t ha ^−1^) under controlled irrigation in the Taihu Lake region, China mitigated both CH _4_ and N _2_O emissions by 35.7% and 21.5%, respectively, and simultaneously enhanced rice yield by 16.7-24.3%. Moreover, Wu *et al.* (2019b) reported that biochar additions (20 - 40 t ha ^−1^) significantly decreased CH _4_ and N _2_O fluxes by 11.2-17.5% and 19.5-26.3%, respectively, and increased grain yield by 7.9-9.2%. In line with our findings, a long-term biochar application (5 – 10 t ha ^−1^) in China's typical double rice plantation region also significantly decreased CH _4_ flux by 26.18% ( Qin *et al.* 2016). Nevertheless, Wang *et al.* (2011) reported that the biochar incorporation (50 ton ha ^−1^) into the soil significantly decreased N _2_O flux by 41.4-93.5% in lab-scale experiments. In parallel, a meta-analysis based on 30 studies with 261 experimental treatments (lab-scale and pilot-scale) from 2007 to 2013 demonstrated that the addition of biochar reduced N _2_O emissions by 54% ( Cayuela *et al.* 2014). However, Koyama *et al.* (2015) reported that biochar application (10-40 t ha ^−1^) reduced CH _4_ flux but did not N _2_O. In the case of Liu *et al.* (2014), biochar supplementation (24-48 t ha ^−1^) significantly reduced CH _4_ flux by 33.9-40.2%, while N _2_O flux significantly increased by 150 to 190%. Overall, biochar amendment could reduce CH _4_ flux from a rice paddy field, but in some cases, the effect on N _2_O flux remains uncertain. Our study demonstrated that rice husk and melaleuca biochar applications with a range of 5-10 t ha ^−1^ significantly reduced both CH _4_ and N _2_O fluxes within a Thionic Glycesol soil in the VMD. Albeit, the biochar application rate between 5 and 10 t ha ^−1^ hardly obtained the disparity of CH _4_ and N _2_O emissions. Thus, a wide range of biochar application amounts should be evaluated to provide more tailored recommendations.

The CH _4_ mitigation by biochar application consistently pertains to the increasing soil oxidation rate and methanotrophs community. Although we did not determine the number of methanogens and methanotrophs, Nan *et al.* (2021) demonstrated that biochar application stimulates the abundance in either methanogens or methanotrophs, with a high amount of methanotrophs detected in most cases resulted in decreasing of CH _4_ flux. Moreover, Wu *et al.* (2019a) reported that biochar applications to fertilized paddy field soils increased the total type I *pmo*A (preferred the CH _4_ environment) and type II *pmo*A (more dynamic in low CH _4_ conditions) methanotrophs compared to non-amended biochar, indicating that CH _4_ flux mitigation by promoting potential CH _4_ oxidation. Thus, we adopted a hypothesis that the balance of activities between methanogens and methanotrophs in a site-specific environment results in either an increasing or decreasing CH _4_ flux. Feng *et al.* (2012) revealed the main mechanisms of CH _4_ flux reduction in a biochar-supplemented field were by (1) increased methanotrophic proteobacterial abundance significantly and (2) decreased the methanogenic to methanotrophic proportion substantially. Thus, an increase of methanotrophs dynamic in paddy field soil by biochar addition can be expected to play a vital role in mitigating CH _4_ fluxes. Our study demonstrated that rice husk and melaleuca biochar could promote low-GHG emissions in the rice production system in the VMD.

We achieved N _2_O flux reduction by incorporating biochar into the topsoil layer when compared to the non-amended biochar field. However, several hypotheses supposed that soil applied with biochar could not decrease the N _2_O flux ( Koyama *et al.*, 2015; van Zwieten *et al*., 2010). Similar to our field study, several findings achieved a total N _2_O flux reduction ( Shaukat *et al.*, 2019; Zhang *et al*., 2010). The mitigation of N _2_O flux in biochar-treated soils could be attributed to soil moisture contents and nitrification processes ( Ameloot *et al.*, 2016). In agreement with the hypothesis, Shaukat *et al.* (2019) demonstrated that fields with biochar added retained 9-14% higher moisture contents than fields without biochar amended and resulted in a significant reduction of the N _2_O flux. Supporting the idea, Wang *et al.* (2013) revealed the relationship between the denitrifying community and N _2_O flux change, where biochar supplementation significantly shifted the abundance of NO _3_-utilizing bacteria (carrying the *nir*K and *nir*S genes), leading to less N _2_O generation and more N _2_O-consuming bacteria (carrying the *nos*Z gene). Moreover, Cayuela *et al.* (2013) used ^15^N gas-flux to observe the reduction of N _2_O/(N _2_+N _2_O) and demonstrated that biochar facilitated the last step of denitrification. The key mechanisms of N _2_O flux reduction under biochar amendment were by (i) stimulated nitrification generation via electron donation, a decrease in total denitrification by serving as an alternative electron acceptor by acting as electron shuttle to soil NO _3_^−^ consuming microorganisms ( Cayuela *et al.*, 2013), and (ii) based on the entrapment of N _2_O in water-saturated soil pores and co-occurrent stimulation of microbial N _2_O reduction deriving in an overall decrease of the N _2_O/(N _2_O + N _2_) ratio ( Harter *et al.*, 2016). Therefore, biochar could be attributed as a decisive factor to inhibit N _2_O production and simultaneously stimulate N _2_O utilization. As such, these findings and the above-discussed mechanisms strongly support our findings in suggesting N2O flux reduction from biochar amendment in the rice paddy field.

Our study showed that N _2_O emission was mainly concentrated during fertilization, which indicates fertilization provides more available N driving for soil N _2_O emission. Xie *et al.* (2013) observed ^15^N abundance significantly intensified by the application of ^15^N-enriched urea. Our study did not measure NH _4_^+^ or NO _3_^−^ concentration during fertilizing, so the mechanism remains uncertain. N _2_O emission via the nitrification process directly pertains to soil physical, chemical, and biological properties ( Huang *et al.*, 2019). Thus, we speculate that N fertilizing increased the nitrification activities and stimulated the strong metabolism of potential N _2_O-producing bacteria. Minamikawa *et al.* (2021) reported that higher N availability levels in soil than rice plant uptake demands resulted in increasing N _2_O emissions. Although N-fertilizing obviously promoted N _2_O emissions for the majority of the time, N _2_O emission peaks of biochar-amended soil were lower than that of biochar-unamended soil. This would indicate that biochar potentially changed the functionality and diversity of denitrifiers within the soils and inhibited the conversion of NO _2_^−^ and NO _3_^−^ to N _2_O ( Zhang *et al.*, 2010).

Water management is a crucial factor in the strategy of GHGs reduction, although we achieved the GHGs reduction under typical water management when most of the time the soil was flooded. Multiple-flooded times in this study were due to the combination of high rainfall in the transition season (rainfall, Figure 2; water level, Figure 3) and the typical flooding water management practice of the farmers in the region. Uno *et al*. (2021) conducted a 2-year field experiment in An Giang province in the VMD and demonstrated that AWD (known as multiple drainages) significantly reduced CH _4_ by 35%, while found no difference in N _2_O emissions, but a 22% yield improved. Moreover, Minamikawa *et al.* (2021) registered that the intermittent irrigation technique is also a promising approach to mitigate CH _4_ emissions by reductive soil conditions. Thus, integrating AWD and intermittent irrigation by incorporating biochar into the soil under the MD’s edaphology, climate, and traditional practices could be feasible for further works.

### Relationship between biochar amendment ratios and CH _4_ and N _2_O fluxes

There is a negative correlation between CH _4_ flux and RhB application rate ( *P <* 0.001, *r* ^2^ = 0.825) ( Figure 4). It is indicated that CH _4_ flux decreased with the increase of rice-husk biochar application ( Xiao *et al.* 2018). On the other hand, although increasing MB application rate could mitigate the CH _4_ emission, the relationship found a poor explanation ( *P =* 0.095, *r* ^2^ = 0.254). This contrast could be partly attributed to biochar-carbonized properties. MB was low in the specific surface area and total pore volume compared to RhB ( Table 1). Ji *et al.* (2020) revealed that biochar structure intimately related to anaerobic CH _4_ oxidation and created a suitable environment for CH _4_-consuming bacteria.

Similarly, we found a negative correlation between the N _2_O flux reductions and the application rate of RhB ( *P =* 0.012, *r* ^2^ = 0.619) and MB ( *P =* 0.002, *r* ^2^ = 0.757). In agreement with our finding, a meta-analysis of Cayuela *et al.* (2014) showed a negative relationship between biochar application rates and reduced N _2_O flux, where sufficient N _2_O reduction was 1-2% biochar amendments, whereas, incorporating more than 10% of biochar into the soil was found to reach up to 80%. In line with our study, Huang *et al.* (2019) also showed a negative relationship between biochar application rates and N _2_O flux. Overall, the increase of biochar application rates could potentially stimulate the CH _4_ and N _2_O reduction. However, for CH _4_ and N _2_O fluxes, the application of 5 and 10 t ha ^−1^ remains unclear.

### Effect of biochar incorporation on Soil Eh and CH _4_ emission

Our study found that the negative linear relationship between soil Eh and hourly CH _4_ flux with RhB ( *P <* 0.001; *r* ^2^ = 0.552) and MB ( *P <* 0.001; *r* ^2^ = 0.502) ( Figure 5). Similar results were also observed by Wang *et al*. (2018). This indicates that an increase of soil redox potential decreased CH _4_ emission, which is in line with the report by Towprayoon (2020). Moreover, soil Eh remained below −250 from 17 to 66 DAS in our study ( Figure 4), implying a favorable condition for CH _4_ emission ( Wang *et al.* 1993). Final drainage rapidly increased soil Eh and reduced CH _4_ flux ( Figure 3), indicating the strong sensibility of soil Eh and CH _4_ flux under water management.

Biochar application increased soil Eh compared to non-amended soils ( Table 4). This indicates that biochar was the critical factor contributing to the positive effects of anaerobic CH _4_ oxidation activities known as the electronic accepting capacities (EAC) of biochar ( Nan *et al.*, 2021). The supplementation of biochar intensifies oxygen-containing functional groups (carboxyl, carbonyl, quinone phenolic hydroxyl group) and positively improves biochar redox potential ( Klüpfel *et al.* 2014; Wu *et al.* 2016). The increase of Eh and the reduction of CH _4_ emissions could also be explained by the porosity and absorbability characteristics of biochar, which enable robust CH _4_-utilizing bacteria activities and intensify the diffusion and metabolism process. In a similar way, biochar incorporation into soils improves soil aeration, creating a favorable environment for methanotrophic bacteria resulting in soil Eh amelioration and better reduction of CH _4_ oxidation ( Feng *et al.*, 2012).

### Effects of biochar incorporation on grain yield, GWP and Yield-scaled GWP

Although biochar amendments could improve yield (2.41-4.21%) ( Table 3), multiple comparison analyses found no significant difference between amended and unamended soils. Several studies have found similar results ( Qin *et al.* 2016; Nguyen *et al.* 2016). The undistinctive grain yield could be partly attributed to spatial and temporal variations, i.e., climatic conditions, field practices, soil substrates ( Xie *et al.* 2013).

Biochar-amended soil significantly decreased GWP by 21.1-30.0% and yield-scaled GWP by 24.5% - 31.8% ( Table 3). It was indicated that RhB and MB application potentially mitigates total CH _4_ and N _2_O emissions without scarifying grain yield. Yang *et al.* (2019) performed a double-season field experiment on biochar applications ranging from 20 to 40 t ha ^−1^ and found that the average GWP and yield-scaled GWP reduced by 18.7% - 16.4%, and 80.3% - 41.6%, respectively. Similarly, Zhang *et al.* (2019) observed a six-year field experiment on biochar-applied soils at rates of between 20-40 t ha ^−1^ and showed a GWP and yield-scaled GWP reduction by 12.1-18.4% and 35.9-56.4%, respectively. Here we observed that CH _4_ flux was the key contributor in the GWP and yield-scaled GWP via the field experiment in the VMD’s transition season, while N _2_O flux was more neglectable. Thus, future works should emphasize on reducing the GWP, yield-scaled GWP, and concentrate on the CH _4_ mitigation technology solutions rather than N _2_O emissions.

### Effects of biochar incorporation on soil improvement

Soil improvement under short-term and long-term biochar applications has been widely recognized. Our study showed that biochar amendment insignificantly increased soil pH ( Table 4), which indicated no effect of biochar addition on soil pH perfection as suggested by previous studies ( Yang *et al.*, 2019). However, biochar amendment significantly decreased the soil bulk density and improved soil porosity in comparison to non-amended soils. Furthermore, higher applied biochar rates showed lesser soil bulk density and higher porosity indicating that biochar directly upgraded soil physiology. Amelioration of soil surface area and porosity by biochar amendment intensifies soil aeration and functions of aeration, such as CH _4_ oxidation, and provides habitat for methanotrophs ( Nan *et al*., 2021). Moreover, it stimulates NH _4_^+^ absorbance ability resulting in suppressing nitrification processes and N _2_O flux reduction in the field ( Wang *et al.*, 2020).

It is evident that increasing biochar application boosted SOM and AN, with a slightly increased available P through the season ( Table 4). The increase of SOM and AN showed a high nutrient availability in the soil. Notably, the soil improvement did not increase soil CH _4_ and N _2_O emissions as above-mentioned and discussed. AN could be used as a soil health indicator ( García *et al.*, 2020). The interdependence among AN, SOC, and particulate OC was demonstrated by a positive correlation ( Domínguez *et al.*, 2016). In connection with our study, Yang *et al*. (2019) observed that biochar amendment slightly increased SOC, significantly increased NH _4_^+^ by 47.7%, and significantly decreased NO _3_^−^ by 30.4%. Incorporating biochar into soils could inhibit nitrification and produce more NH _4_^+^ than NO _3_^−^ consisting of an anoxic environment (water level and redox potential; Figure 2). Increasing NH _4_^+^ concentrations and declining NO _3_^−^ concentrations would partly explain the enhanced CH _4_-consuming figure and N _2_O oxidation ( Xiao *et al.*, 2018). Overall, biochar application offers benefits not only for nutrient availability but also for GHGs mitigation.

## Conclusions

This study assessed the effects of rice husk biochar or melaleuca biochar amendment at 5 or 10 t ha ^−1^ on CH _4_ and N _2_O emissions and the physiochemical soil properties after rice cultivation under typical water management and conventional practice regime in the VMD. Incorporating biochar into soils significantly mitigated CH _4_ and N _2_O emissions without reducing grain yield. Consequently, a lower GWP and yield-scaled GWP from biochar-amended soils were achieved. Although higher biochar applications decreased CH _4_ and N _2_O emissions, there was no significant difference between biochar-amended rates. Biochar significantly increased soil Eh conditions. There was a negative linear relationship between soil Eh and CH _4_ emission rate for biochar-applied fields. N _2_O emissions from biochar fields were relatively low and mostly concentrated during the fertilization period. Biochar amendments improved soil fertility via physical properties of soils by decreasing bulk density and intensifying porosity and the chemical characteristics of the soils by ameliorating SOM, AN and AP, but did not affect soil pH. Similar to GHG emissions, biochar application rates of between 5 and 10 t ha ^−1^ could not obtain significant soil improvement. This study will help lower-GHG emissions from rice farming practices in the VMD. Further works should study the combination of biochar-application rates and effective water irrigation techniques on different soils in the VMD.

## Data availability

### Underlying data

Figshare: Biochar reduces GHGs from paddy fields. https://doi.org/10.6084/m9.figshare.16625137.v1 ( Tran Sy *et al*., 2021).

This project contains the following underlying data:

- Nam et al_Raw data biochar_F1000research.xlsx

Data are available under the terms of the Creative Commons Zero “No rights reserved” data waiver (CC0 1.0 Public domain dedication).
