# Supplementary material for: Rice husk and melaleuca biochar additions reduce soil CH 4 and N 2O emissions and increase soil physicochemical properties
Source: F1000Res. 2022 Feb 15;10:1128. Originally published 2021 Nov 8. [Version 2] doi: 10.12688/f1000research.74041.2 (PMC8881694; doi:10.12688/f1000research.74041.2)
Supplement: Peer review history

# Peer review: Reviewer response for version 2

**DOI:** 10.5256/f1000research.121175.r123613

**Published:** 2022-02-25

**Authors:** Azeem Tariq

**Affiliations:** 1 Department of Plant and Environmental Sciences, University of Copenhagen, Copenhagen, Denmark

No further comments.

Is the work clearly and accurately presented and does it cite the current literature?

Yes

If applicable, is the statistical analysis and its interpretation appropriate?

Partly

Are all the source data underlying the results available to ensure full reproducibility?

Partly

Is the study design appropriate and is the work technically sound?

Yes

Are the conclusions drawn adequately supported by the results?

Yes

Are sufficient details of methods and analysis provided to allow replication by others?

Yes

Reviewer Expertise:

Climate change mitigation, GHG emissions, sustainable crop rotations, ecosystem modeling

I confirm that I have read this submission and believe that I have an appropriate level of expertise to confirm that it is of an acceptable scientific standard.

# Peer review: Reviewer response for version 2

**DOI:** 10.5256/f1000research.121175.r123614

**Published:** 2022-02-21

**Authors:** Bui Truong Tho

**Affiliations:** 1 The Centre of Hi-tech Application in Agriculture, Chau Thanh district, Vietnam

The authors have revised the article and it is better than previous one. I totally agree with the new version.

Is the work clearly and accurately presented and does it cite the current literature?

Yes

If applicable, is the statistical analysis and its interpretation appropriate?

Partly

Are all the source data underlying the results available to ensure full reproducibility?

Partly

Is the study design appropriate and is the work technically sound?

Yes

Are the conclusions drawn adequately supported by the results?

Yes

Are sufficient details of methods and analysis provided to allow replication by others?

Yes

Reviewer Expertise:

Hi-tech Application in Agriculture, Climate change mitigation, GHG emissions, Sustainable crop rotations, Plant ecophysiology.

I confirm that I have read this submission and believe that I have an appropriate level of expertise to confirm that it is of an acceptable scientific standard.

# Peer review: Reviewer response for version 1

**DOI:** 10.5256/f1000research.77750.r120157

**Published:** 2022-02-08

**Authors:** Bui Truong Tho

**Affiliations:** 1 The Centre of Hi-tech Application in Agriculture, Chau Thanh district, Vietnam

This article, "Rice husk and Melaleuca biochar additions reduce soil CH _4_ and N _2_O emissions and increase soil organic matter and nutrient availability" aims to evaluate the impacts of two biochar amendments on soil CH _4_ and N _2_O emissions and its effect on soil physiochemical properties in Mekong Delta based on 80 days of field study.

 The topic is interesting and poorly treated in literature. The data are correctly processed and the results are well discussed. However, there are some minor issues and my reviews are focused on improving a revision of the current article, and I hope the authors can accommodate my suggestions.

1. Site description: “air temperature (27.5 – 27.5 °C)” I think there is a mistake, authors should double-check the information.
2. Statistical analysis: Authors should confirm that all data were test homogeneity of variance for using One-Way analysis of variance (ANOVA).
3. Authors used “Vietnamese Mekong Delta (VMD)”, some places used “Mekong Delta (MD)”, it should unified in all the manuscript.

Is the work clearly and accurately presented and does it cite the current literature?

Yes

If applicable, is the statistical analysis and its interpretation appropriate?

Partly

Are all the source data underlying the results available to ensure full reproducibility?

Partly

Is the study design appropriate and is the work technically sound?

Yes

Are the conclusions drawn adequately supported by the results?

Yes

Are sufficient details of methods and analysis provided to allow replication by others?

Yes

Reviewer Expertise:

Hi-tech Application in Agriculture, Climate change mitigation, GHG emissions, Sustainable crop rotations, Plant ecophysiology.

I confirm that I have read this submission and believe that I have an appropriate level of expertise to confirm that it is of an acceptable scientific standard.

# Response

**Published:** 2022-02-09

**Authors:** Thao Huynh Van

**Affiliations:** College of Environment and Natural Resources, Cantho Univeristy, Vietnam

Thank you very much for your constructive comments. All your comments have been carefully read and revised by the authors. Our responses are enclosed as follows:

**Comment 1#.** Site description: “air temperature (27.5 – 27.5 °C)” I think there is a mistake, authors should double-check the information.

***Reply 1:*** Thank you for finding this mistake. We re-referred to the original document and corrected the information in the manuscript. The air temperature should be 27.5 ^o^C instead.

**Comment 2#:** Statistical analysis: Authors should confirm that all data were test homogeneity of variance for using One-Way analysis of variance (ANOVA).

***Reply 2:*** We amended additional information related to the homogeneity of variance in the statistical analysis. The revised is as follows “Significant different comparison among treatments was considered at Duncan’s multiple range test (***P < 0.001, **P < 0.01, *P < 0.05 and †P > 0.05) after passing homogeneity of variance”.

**Comment 3:** Authors used “Vietnamese Mekong Delta (VMD)”, some places used “Mekong Delta (MD)”, it should be unified in all the manuscript.

***Reply 3:*** We have unified the abbreviation in all parts of the manuscript which is “VMD”.

 Best regards,

 Authors.

# Peer review: Reviewer response for version 1

**DOI:** 10.5256/f1000research.77750.r99426

**Published:** 2022-01-04

**Authors:** Azeem Tariq

**Affiliations:** 1 Department of Plant and Environmental Sciences, University of Copenhagen, Copenhagen, Denmark

The authors of "Rice husk and melaleuca biochar additions reduce soil CH _4_ and N _2_O emissions and increase soil organic matter and nutrient availability" present an effort in assessing the effects of two biochar amendments on soil CH _4_ and N _2_O emissions and effects on soil physiochemical properties based on 80 days of field study. The overall paper has been written in a good English and in a logical manner. However, there are some minor issues that authors need to deal before considering this paper for indexing.

 Authors used the term “soil organic matter and nutrient availability” in title, but they have focused on different soil properties e.g. soil porosity, soil bulk density, soil redox potential, soil organic matter and nitrogen. Authors should use the term soil physiochemical properties instead.

 Authors did not explain the results related to soil properties in the abstract section (e.g. there is no explanation about soil organic matter and nitrogen contents in abstract).

 Authors stated that they used 95 % confidence level, but the result section predicted that authors have used different levels of probability for difference analysis. Author should explain the analysis carefully in the statistical analysis section in Material and Methods.

Is the work clearly and accurately presented and does it cite the current literature?

Yes

If applicable, is the statistical analysis and its interpretation appropriate?

Partly

Are all the source data underlying the results available to ensure full reproducibility?

Partly

Is the study design appropriate and is the work technically sound?

Yes

Are the conclusions drawn adequately supported by the results?

Yes

Are sufficient details of methods and analysis provided to allow replication by others?

Yes

Reviewer Expertise:

Climate change mitigation, GHG emissions, sustainable crop rotations, ecosystem modeling

I confirm that I have read this submission and believe that I have an appropriate level of expertise to confirm that it is of an acceptable scientific standard, however I have significant reservations, as outlined above.

# Response

**Published:** 2022-02-09

**Authors:** Thao Huynh Van

**Affiliations:** College of Environment and Natural Resources, Cantho Univeristy, Vietnam

Thank you very much for giving us constructive comments that helped us improve this paper immensely. All your comments have been carefully read and revised to elucidate underlying aspects. Our responses to your comments are as follows:

**Comment 1:** Authors used the term “soil organic matter and nutrient availability” in title, but they have focused on different soil properties e.g. soil porosity, soil bulk density, soil redox potential, soil organic matter and nitrogen. Authors should use the term soil physiochemical properties instead.

***Reply 1#:*** Thank you very much for the recommendation for the title term of “soil physiochemical properties”. The recommendation has been adopted. The revision is as follows “Rice husk and melaleuca biochar additions reduce soil CH _4_ and N _2_O emissions and increase soil physicochemical properties.”

**Comment 2:** Authors did not explain the results related to soil properties in the abstract section (e.g. there is no explanation about soil organic matter and nitrogen contents in abstract).

***Reply 2#:*** We revised the abstract with additional information related to the explanation and implication for improving soil physiochemical characteristics. The revision is as follows “The results showed…Ameliorating soil aeration and functions by adding RhB and MB resulted in improving soil physicochemical characteristics, especially significant SOM and AN boosting, which indicate better soil health, structure, and fertility”.

**Comment 3:** Authors stated that they used 95% confidence level, but the result section predicted that authors have used different levels of probability for difference analysis. Author should explain the analysis carefully in the statistical analysis section in Material and Methods.

***Reply 3#:*** We added more information to the statistical level to make it clearer to readers. The supplementations are as follows “The results are…Significant different comparison among treatments was considered at Duncan’s multiple range test (***P < 0.001, **P < 0.01, *P < 0.05 and †P > 0.05)”

 We look forward to hearing from you.

 Best regards,

 Authors.
